# Total and Added Sugar Intake: Assessment in Eight Latin American Countries

**DOI:** 10.3390/nu10040389

**Published:** 2018-03-22

**Authors:** Mauro Fisberg, Irina Kovalskys, Georgina Gómez, Attilio Rigotti, Lilia Yadira Cortés Sanabria, Martha Cecilia Yépez García, Rossina Gabriella Pareja Torres, Marianella Herrera-Cuenca, Ioná Zalcman Zimberg, Berthold Koletzko, Michael Pratt, Luis A. Moreno Aznar, Viviana Guajardo, Regina Mara Fisberg, Cristiane Hermes Sales, Ágatha Nogueira Previdelli, on behalf of the ELANS Study Group

**Affiliations:** 1 Instituto Pensi, Fundação Jose Luiz Egydio Setubal, Sabará Hospital Infantil, São Paulo 01239-040, Brazil; 2 Departamento de Pediatria, Escola Paulista de Medicina, Universidade Federal de São Paulo, São Paulo 04023-062, Brazil; 3 Committee of Nutrition and Wellbeing, International Life Science Institute (ILSI-Argentina), Buenos Aires C1059ABF, Argentina; ikovalskys@gmail.com (I.K.); vbguajardo@gmail.com (V.G.); 4 Departamento de Bioquímica, Escuela de Medicina, Universidad de Costa Rica, San José 11501, Costa Rica; georgina.gomez@ucr.ac.cr; 5 Centro de Nutrición Molecular y Enfermedades Crónicas, Departamento de Nutrición, Diabetes y Metabolismo, Escuela de Medicina, Pontificia Universidad Católica, Santiago 833-0024, Chile; arigotti@med.puc.cl; 6 Departamento de Nutrición y Bioquímica, Pontificia Universidad Javeriana, Bogotá 110111, Colombia; ycortes@javeriana.edu.co; 7 Colegio de Ciencias de la Salud, Universidad San Francisco de Quito, Quito 17-1200-841, Ecuador; myepez@usfq.edu.ec; 8 Instituto de Investigación Nutricional, La Molina, Lima 15026, Peru; rpareja@iin.sld.pe; 9 Centro de Estudios del Desarrollo, Universidad Central de Venezuela (CENDES-UCV)/Fundación Bengoa, Caracas 1010, Venezuela; marianella.herrera@ucv.ve; 10 Departamento de Psicobiologia, Universidade Federal de São Paulo, São Paulo 04023-062, Brazil; iona.zimberg@gmail.com; 11 University of Munich Medical Center, Division of Metabolic and Nutritional Medicine, Dr. von Hauner Children’s Hospital, Ludwig-Maximilians-Universität München, D-80337 Munich, Germany; berthold.koletzko@med.uni-muenchen.de; 12 Department of Family Medicine and Public Health, University of California, San Diego, CA 92093, USA; mipratt@ucsd.edu; 13 Facultad de Ciencias de la Salud, Growth, Exercise, Nutrition and Development (GENUD) Research Group, Universidad de Zaragoza, Zaragoza 50009, Spain; lmoreno@unizar.es; 14 Departamento de Nutrição, Faculdade de Saúde Pública, Universidade de São Paulo, São Paulo 03178-200, Brazil; regina.fisberg@gmail.com (R.M.F.); cristianehermes@yahoo.com.br (C.H.S.); 15 Faculdade de Ciências Biológicas e da Saúde, Universidade São Judas Tadeu, São Paulo 03166-000, Brazil; agatha.usp@gmail.com

**Keywords:** cross-sectional study, dietary intake, Latin American, nutrition, sugars, survey

## Abstract

Non-communicable diseases are growing at an alarming rate in Latin America. We assessed total and added sugar intake in Argentina, Brazil, Chile, Colombia, Costa Rica, Ecuador, Peru, and Venezuela, to verify the adequacy of the World Health Organization’s recommendations, considering gender, socioeconomic level (SEL) and age. A total of 9218 non-institutionalized individuals living in urban areas (age range 15–65 years) were assessed in the Latin American Study of Nutrition and Health (ELANS), a multicenter household population-based cross-sectional survey. Socio-demographic data were collected. Total and added sugar intakes were measured using two non-consecutive 24-h dietary recalls. The prevalence of excessive sugar intake was estimated. A large proportion of individuals showed high consumption of total and added sugar intake, which reflected in the high prevalence of excessive sugar intake. With minimal differences across countries, in general, women, individuals with high SEL, and younger people had higher percentages of total energy intake from total and added sugar intake, and of contribution of carbohydrates from total and added sugars. Thus, there is high consumption of total and added sugar intake in the Latin American countries with some peculiarities considering socio-demographic variables, which should be considered in each country’s health intervention proposals.

## 1. Introduction

In previous centuries, sugar was used as a commodity currency and represented wealth [1,2]. In this way, it was common to use high amounts of sugar in the food preparations to demonstrate possessions, and this influenced the taste preferences as a result of experiential learning that was passed down the generations [1,3]. On the other hand, sugar is traditionally used as preservative of foods, just as salt [1].

Nowadays, despite some studies having shown that sugar intake is decreasing [4,5,6,7], globally a high sugar intake is often seen [4,8,9,10,11,12,13,14,15,16]. In epidemiological studies developed around the world, total sugar was observed ranging from 14.5 to 21.9% of total energy intake (%TE) in adults of both genders assessed in Italy [9] and individuals of both genders, 18–34 years, assessed in National Health and Nutritional Examination Survey III in the United States of America [4]. Furthermore, for added sugar ranges from 7.2 to 21.4%TE were observed in men, 18–70 years, assessed in Norkost 3, Norway [4], and individuals of both genders, 18–34 years, assessed in the National Health and Nutritional Examination Survey in the United States of America [4].

With the worldwide increase in non-communicable disease (NCD) epidemics, and the known association of sugar intake with health implications [14,17,18], the global health agenda has been drawn to public health policies with the purpose of awareness in the population and the private sector to the emerging need to reduce, mainly, added sugar consumption [19]. More recently, the World Health Organization (WHO) has highlighted the consumption of free sugar, referred to as the sum of added sugar, sugars naturally present in honey, syrups and fruit juices and fruit juice concentrates, and whose consumption has been associated with poor dietary quality, besides higher risk of developing dental caries, obesity, and NCD [19]. However, despite some authors having attempted to show the extent of free sugar intake [10,16,20], the assessment is globally limited because of the lack of databases on free sugar in the national tables of nutritional composition, and by the lack of a universal standardized definition [20,21,22].

Despite the difficulty of evaluating added sugar, and particularly free sugar, the reporting of sugar intake can guide adoption of public policies, and it is suggested that this information must be monitored more rigorously, especially in countries whose data from sugar intake are scarce, which includes most Latin American countries, a region of the south American continent characterized by a conglomerate of developing countries that experience a dual scenario referred to as nutritional transition. In addition, it is important to identify factors such as gender, socioeconomic level, and age that may influence dietary habits and contribute to a higher sugar intake [8,23,24,25]. This will facilitate the planning of action strategies targeting the most vulnerable groups.

Given this background, this study aimed to assess the total and added sugar intake in a representative sample of Latin American countries, and to verify the adherence to the WHO recommendations, considering gender, socioeconomic level (SEL), and age range.

## 2. Materials and Methods

This survey was approved by the Western Institutional Review Board (#20140605), by the ethics review boards of the participating institutions, and is registered at Clinical Trials (#NCT02226627). Written informed consent/assent was obtained from all individuals before commencement of the study. Participant confidentiality for the pooled data was maintained via the use of numeric identification codes rather than names. All data transfer was done with a secure file sharing system.

### 2.1. Study Population

This study examined data from the Latin American Study of Nutrition and Health (‘*Estudio Latinoamericano de Nutrición y Salud*’; ELANS), a household-based multi-national cross-sectional population-based survey, conducted from March 2014 to December 2015 in eight Latin American countries: Argentina, Brazil, Chile, Colombia, Costa Rica, Ecuador, Peru, and Venezuela.

Sample recruitment was performed using a random complex, multistage sampling. Individuals (*n* = 9218; aged 15 to 65 years) were stratified by geographical location (only urban areas), gender, age, and SEL. Only urban areas were included considering that almost all countries included in ELANS have at least 80–90% of the population living in urban areas. The sample size was calculated using a confidence level of 95% and a sample error of 3.49% at a 5% significance level and survey design effect of 1.75. More details can be seen in Fisberg et al. [26].

Pregnant and lactating women (in the first six months postpartum), individuals with major physical or mental impairments that affect food intake or physical activity, individuals outside of age range 15–65 years, adolescents without assent or consent of a parent or legal guardian, individuals living in institutions, and individuals unable to read were not included in the sample.

### 2.2. Data Entry and Databases

Trained interviewers visited the selected households twice. At the first visit, the ELANS’ purpose was explained, and the eligible individuals signed the informed consent/assent form. Thereafter, a structured questionnaire was used to collect information about demographics (age, gender, years of education, number of people in the household, race/ethnicity, marital status, and number of years living in the country) and SEL. A 24-h dietary recall (24-HR) was applied, and anthropometric measurements were assessed. At the second visit, a second 24-HR and a beverage intake questionnaire were answered.

Gender was classified as male or female. Age on the date of interview was considered in complete years, and the individuals were stratified into four age groups: 15–19, 20–34, 35–49, and 50–65 years. SEL was classified into high, medium, and low strata, and these were based on the national indexes used in each country, as described in Fisberg et al. [26].

The 24-HR were collected in nonconsecutive days, including weekend days, by trained interviewers who used a structured script elaborated in paper form to be used in the home interviews. The United States Department of Agriculture’s (USDA) five-step multiple-pass method [27] was followed during the interviews to guide the individuals and to facilitate in recalling the foods and beverages consumed. This method is composed by the following steps: 1. quick list—the interviewee lists, without interruption, all foods and beverages consumed the previous day; 2. forgotten foods list—the interviewer repeats the list of foods and beverages mentioned by the interviewee to identify foods that may have been forgotten; 3. time and occasion—the interviewee elaborates on the time he/she consumed foods and on what he/she considers to be a meal; 4. detail cycle—the interviewee provides descriptions and amounts of each food reported, and the interviewer reviews each occasion and the interval between occasions; 5. final review probe—the interviewer repeats all information with the intention of collecting data on additional foods not remembered earlier. The data were analyzed using the Nutrition Data System for Research software version 2014 (NDS-R), a dietary assessment tool developed by the Nutrition Coordinating Center of University of Minnesota, Minneapolis, MN, whose steps to 24-HR data insertion follow the five-steps multiple pass method. Further details about the food matching standardized procedures can be seen elsewhere [28].

### 2.3. Total and Added Sugar Intakes

Information on total and added sugar intakes were obtained from the 24-HR analysis using the NDS-R, that includes in the 2014 version the USDA’s database, data from scientific literature, food manufactures, and foreign food composition tables, including Latin American Food Composition Table. In this software database, information on total sugar is captured and analyzed as being composed of the individual intrinsic and extrinsic monosaccharides (glucose, fructose and galactose) and disaccharides (sucrose, lactose and maltose); and added sugar (by total sugars) by those sugars and syrups added to foods during food preparation or commercial food processing (white sugar (sucrose), brown sugar, powdered sugar, honey, molasses, pancake syrup, corn syrups, high fructose corn syrups, invert sugar, invert syrup, malt extract, malt syrup, fructose, glucose (dextrose), galactose, and lactose), excluding mono- and disaccharides occurring naturally in foods, such as lactose in milk or fructose in fruit [29].

When necessary, sugar and added sugar content were corrected to approximate the local reality using a data correction routine made to be used in the Stata software (version 13.0; StataCorp LP, College Station, TX, USA), and a corrected database was generated.

The prevalence of individual excessive added sugar intake was determined using the WHO’s recommendations intake of free sugars—below 10%TE—and the WHO’s conditional recommendations on intake of free sugars—below 5%TE [19].

### 2.4. Statistical Analyses

Usual dietary intake of sugars was estimated using the Multiple Source Method, a web-based tool developed by researchers at the European Prospective Investigation into Cancer and Nutrition (EPIC) for estimating usual dietary intakes of nutrients and foods consumed by populations and individuals, available at https://msm.dife.de/. The method is accomplished in three steps which requires at least two days of dietary measurements on a random subsample of the target population. First, the probability of nutrient/food intake for each individual is estimated using logistic regression with random effects (probability model). Second, the usual amount of nutrient/food intake in days of consumption is estimated using linear regression, also with random effects (quantity model). Finally, the individual’s usual nutrient/food intake is calculated by multiplying the result of the probability of nutrient/food intake obtained in the first step with the usual amount of nutrient/food intake obtained in the second step [30].

Total and added sugar intake were presented as means, standard deviation, percentiles, %TE, and percentage of carbohydrate contribution (%CHO). The percentage of individuals with excessive added sugar intake was shown as columns in the figures. Stata software was used to perform these statistical analyses, and data were stratified by gender, SEL, and age groups, and considered in the set of the countries assessed in ELANS and individually. The proportions of sugar adequacy were compared using the Mann–Whitney or Kruskal–Wallis test, after the normality had been tested by the Skewness and Kurtosis test. The level of significance was established at 5%.

## 3. Results

The mean total sugar intake for all countries was 99.4 g/day, which accounted for 20.1% of total energy (%TE) and contributed to 36.7% of the total carbohydrates consumed (Table 1). Of total sugar intake, 65.9% was coming from added sugar intake, which contributed to 13.2%TE (65.5 g of added sugar/day; 23.9% of the total carbohydrates; Table 2). Comparing the countries, in absolute terms (g/day), Argentina, Colombia, and Peru had the highest values of total sugar intake. In these three countries, as well as Costa Rica and Venezuela, there was a greater contribution of total sugar intake to the energy intake in relative terms (%TE) compared to the other countries. On the other hand, Brazil and Chile had the lowest values of total sugar intake in absolute terms, and Brazil and Ecuador in relative terms (Table 1). With respect to added sugar intake, the values in Argentina were considerably higher than the other countries in absolute (g/day) and relative terms (%TE and % of sugar from total carbohydrates; %CHO), whereas the lowest values were observed in Chile in absolute terms, and in Ecuador in relative terms (%TE and %CHO) (Table 2).

In general, men had consumed larger amounts of total and added sugar in absolute terms (mean g/day), a finding that was consistent among the countries when assessed separately (Table 3 and Table 4). However, in relative terms, the %TE and %CHO showed that women had the highest values in all countries with respect to total sugar intake (Table 3) and in most countries with respect to added sugar intake (Table 4).

Considering the SEL, the total sugar intake in absolute and relative terms of all countries was greater among individuals in the high strata compared to the others (Table 5); however, there were no differences in added sugar intake among the strata of SEL (Table 6). When the countries were considered independently, the profile observed for all countries was maintained for the majority of the countries (Table 5 and Table 6).

According to age groups, total and added sugar intakes showed a decrease with advancing age, markedly in the absolute terms (Table 7 and Table 8). However, when the amount of total sugar intake was expressed relative to total energy intake (%TE), the differences among age groups were observed only for Brazil and Chile. For added sugar intake, more countries showed differences, being similar between groups for Colombia, Ecuador, Peru, and Venezuela (Table 7 and Table 8).

**Figure 1:**
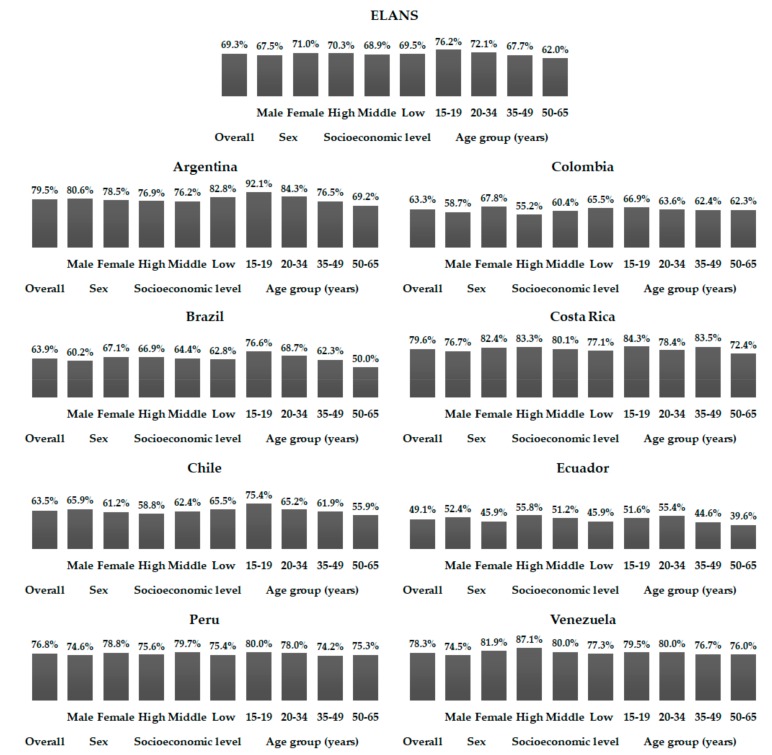
Percentages of individuals with added sugar intake up to 10% of the total energy intake among Latin American countries, according to gender, socioeconomic level, and age ranges; Latin American Health and Nutrition Study (ELANS), 2015.

**Figure 2:**
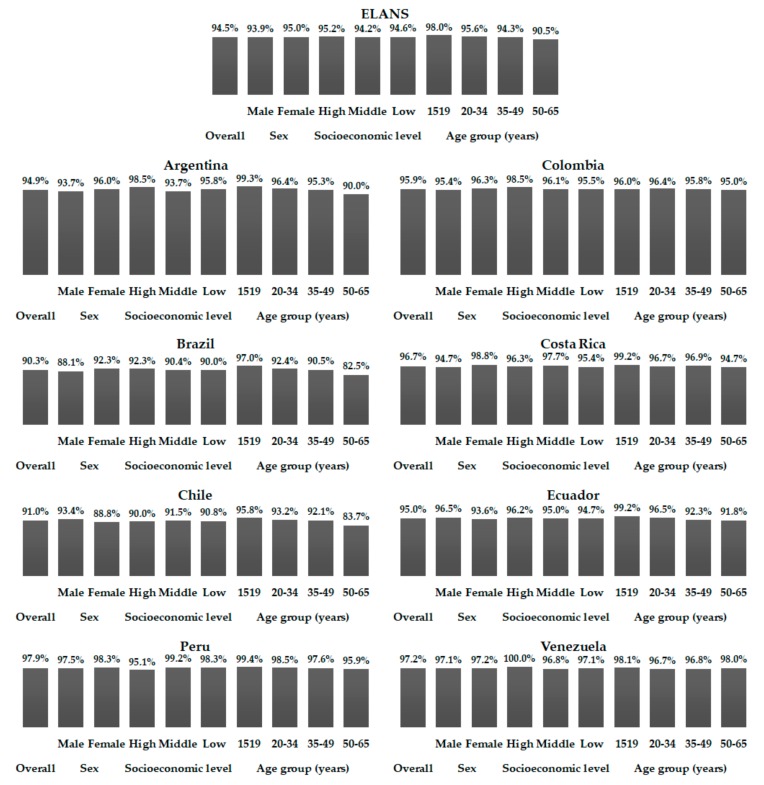
Percentages of individuals with added sugar intake up to 5% of the total energy intake among Latin American countries, according to gender, socioeconomic level, and age ranges; Latin American Health and Nutrition Study (ELANS), 2015.

As shown by means of %TE from added sugar in Table 1, Table 2, Table 3, Table 4, Table 5, Table 6, Table 7 and Table 8, a high percentage of individuals had excessive added sugar intake (Figure 1 and Figure 2). Considering the 10%TE recommendation, approximately 70% of all individuals showed added sugar intake above 10%TE, ranging from 49.1% in Ecuador to 79.6% in Costa Rica. Considering the stratifications performed (gender, SEL, and age), minimal differences were observed in relation to the data presented in the tables among the countries independently (Figure 1). When the 5%TE recommendation was considered, the percentage of excessive added sugar intake leapt to more than 90%, varying among countries from 90.3% in Brazil to 97.9% in Peru, and again showed only slight differences to the general standard when countries were analyzed individually (Figure 2).

## 4. Discussion

This is the first multinational nutrition survey of a representative sample of Latin American countries. It showed a considerable percentage of individuals with a high intake of total and, mainly, added sugar in the countries assessed in ELANS. In addition, in most countries assessed, total sugar intake, in particular, has been influenced by gender, SEL and age.

Sugars occur naturally in foods and is one source of calories which provide energy for the bodily functions [6]. Mainly added sugars overconsumption is frequently associated with reduced diet quality [31,32,33], and it has an important pathophysiological role, predisposing to critical cardiometabolic effects, including weight gain, diabetes, and cardiovascular disease outcomes [17,34,35,36,37] which negatively impact on the prevalence of disease and mortality, disability-adjusted life years and costs averted [38]. Although some countries have shown that sugar intake is decreasing [4,5,6,7], the total amounts consumed are still high considering the current recommendations, as demonstrated in the present study. There is a discussion about which type of sugar is unhealthier, intrinsic or added sugars [39,40,41], and for this reason its consumption should be used as a sentinel, or if the concern should not be sugar itself but the set of unbalanced factors associated with high sugar intake [39,40,42] is an important reason why sugar intake should be monitored.

On the other hand, an absence of a standardized global definition for added and free sugars ends up failing on recommendations for sugar intake and makes it difficult to compare the intake of sugars among the studies. Some countries and societies have suggested several recommendations based on their own definition for added/free sugar; however, the WHO’s recommendations are the most widely used, and for this reason we opted to use it. Furthermore, Latin American countries do not have a quantitative recommendation for total or added sugar intakes. WHO’s recommendation for free sugar is relied upon in terms of the effects triggered in adults and children from reduced and increased free sugar intake [36].

Despite the problem of non-standardization, among the countries assessed in ELANS, Argentina was shown to be most vulnerable to high sugar intakes, obtaining the highest values (absolute and relative) compared to the other countries assessed in ELANS [6,7,9] as well as the US adult population [12,43] that is known to have markedly high sugars diets. The other countries assessed in ELANS presented absolute and relative values of sugars intake that, on average, are similar to those observed in the other countries.

These observations highlight the importance of understanding what is different in Argentina. Perhaps the food that contributes to sugar intake may be the answer. Analysis of sugars contributors is already being done and will be presented in another manuscript in a close future. On the other hand, particular characteristics of the population, such as an affinity for sweet taste, and food environment can have an influence in this [3]. It is known that the culture of sugar consumption in Latin American countries is not a novel tendency but a historical fact having been passed on over the generations, and possibly more strongly in Argentina. In addition, factors such as the composition of gut microbiota should be considered, as it has been demonstrated that bacterial composition may exert an influence on the host’s eating behavior by mechanisms independent of taste and flavor which can predispose to craving for sugar foods, for example [44].

When the prevalence high sugar intake by individuals was assessed, Brazil and Chile showed the lowest and Argentina the highest for added sugar only. The high prevalence was maintained only for total sugar. For other perspectives, Argentina basically presented the same behavior when the sample of the countries assessed in ELANS was stratified by some factors.

In present study, as demonstrated elsewhere [7,9,23,24], in absolute terms men showed higher sugar intakes than women; however, in relative terms this observation was the opposite, which reflects the greater energy intake by men inherent in their constitution that demands greater energy needs. In contrast, higher relative values in women demonstrate that they are consuming a more nutritionally unbalanced diet, at least as far as sugar intake is concerned, and it is still shown in studies investigating sugar-intake trends that the decline in sugar intake is lower in women compared to men [7,9].

Further, in present study when it is observed the total of the countries the difference in SEL were observed only for total sugar intake, being the higher intake observed among those with the highest socioeconomic strata. This finding was the opposite seen in US adults studied by Park et al. [23]. This can reflect different tax incentives to decrease added sugar consumption, which permits the acquisition of items that have more sugar by individuals of distinct SEL. Nevertheless, this hypothesis cannot be automatically generalized to Latin American countries. Sugar sources of added sugar in Latin America could be part of regional foods; however, this should be investigated in future studies.

With respect to the influence of age, following the trend observed in other studies [8,23,24,43], younger individuals were seen to be more vulnerable to higher total and added sugars intake, whose consumption decreased over their lifespan. This can be explained by their immaturity in the choice of their foods, and largely by influence of advertisements. The study of food sources might lend greater insights about these findings and provide clear answers to propose a prudent strategy to promote health benefits for the groups that should be monitored more closely.

It is important to emphasize that the WHO’s recommendation was established for free sugar intake [19]; however, due to the difficulty in quantifying it, this recommendation is used as a reference for added sugar, as used in the present study, and recognized as a limitation. Additionally, it is necessary to recognize that the evaluation of dietary intake is subject to random and systematic errors, despite the care taken to minimize them, as presented elsewhere [26,28], and of the adjustment for intra-individual variation. Nevertheless, despite these limitations, the present study provides useful information to be used to develop public policies that may help in healthier food intake and improved quality of life.

## 5. Conclusions

Total and added sugar intakes were consumed in large quantities in Latin American countries, and seem to present a similar pattern among gender, SEL, and age group strata, with minimal differences in some countries. The peculiarities of each country studied should be taken into consideration in the health interventions proposals.

## Figures and Tables

**Table 1:** Total sugar intakes ^1^ in individuals residing in urban areas of Latin American countries; Latin American Health and Nutrition Study (ELANS), 2015.

Country	*n*	Total Sugar Intake														
		g/Day					%TE					%CHO				
		Mean	SD	P25	P50 ^2^	P75	Mean	SD	P25	P50 ^2^	P75	Mean	SD	P25	P50 ^2^	P75
Argentina	1266	115.2	53.2	75.5	107.5 ^a^	145.9	21.1	7.1	16.2	20.8 ^a^	25.5	40.3	10.8	33.3	40.6 ^a^	47.9
Brazil	2000	86.2	39.3	58.5	80.9 ^b^	107.4	19.1	6.5	14.6	19.0 ^b,c^	23.3	37.0	10.5	30.2	37.4 ^b^	44.1
Chile	879	84.9	33.9	60.5	80.0 ^b^	104.8	19.8	5.6	16.0	19.7 ^b^	23.3	36.3	8.5	30.9	36.6 ^b,c^	41.6
Colombia	1230	109.8	34.4	85.7	105.6 ^a^	128.8	20.9	4.8	17.6	20.7 ^a^	23.8	38.4	7.1	33.6	38.5 ^d,e^	43.1
Costa Rica	798	95.8	39.1	68.3	91.5 ^c^	116.1	20.7	6.1	16.6	20.6 ^a^	24.4	35.5	9.1	29.7	35.4 ^c^	41.6
Ecuador	800	102.4	35.1	78.4	98.1 ^d^	120.4	18.7	4.6	15.4	18.6 ^c^	21.7	33.8	7.1	29.1	34.0 ^f^	38.5
Peru	1113	106.4	34.8	82.2	101.8 ^a^	126.1	20.4	4.8	16.9	20.3 ^a^	23.5	31.1	7.0	26.3	31.1 ^g^	36.1
Venezuela	1132	98.8	39.1	70.7	93.0 ^c,d^	122.0	20.7	5.9	16.8	20.8 ^a^	24.5	38.6	8.9	32.8	38.9 ^e^	45.0
**All**	**9218**	**99.4**	**41.0**	**70.9**	**94.6**	**121.0**	**20.1**	**5.9**	**16.2**	**20.0**	**23.8**	**36.7**	**9.4**	**30.5**	**36.8**	**42.8**

g/day: grams per day; %TE: % of the total energy intake; %CHO: % of the total carbohydrates; SD: standard deviation; P25: 25th percentile; P50: 50th percentile (median); P75: 75th percentile. ^1^ Sugar intakes were adjusted by intra-individual variation. ^2^ Within a column, countries followed by different lower-case letters are significantly different according to Kruskal–Wallis test (*α* = 5%).

**Table 2:** Added sugar intakes ^1^ in individuals residing in urban areas of Latin American countries; Latin American Health and Nutrition Study (ELANS), 2015.

Country	*n*	Added Sugar Intake (from Total Sugar)														
		g/Day					%TE					%CHO				
		Mean	SD	P25	P50 ^2^	P75	Mean	SD	P25	P50 ^2^	P75	Mean	SD	P25	P50 ^2^	P75
Argentina	1266	91.4	55.2	51.2	82.4 ^a^	122.9	16.4	7.5	11.1	15.7 ^a^	21.2	31.0	12.2	22.3	31.3 ^a^	39.7
Brazil	2000	57.6	34.1	32.4	52.4 ^b^	76.3	12.6	6.0	8.0	12.2 ^b^	16.4	24.3	10.2	16.9	24.2 ^b^	31.3
Chile	879	52.3	28.4	31.4	47.9 ^c^	68.5	12.0	5.2	8.3	11.8 ^b,c^	15.6	22.0	8.7	16.0	21.9 ^c^	28.2
Colombia	1230	59.5	24.1	42.7	56.6 ^d^	73.5	11.4	4.0	8.7	11.2 ^c^	13.8	20.9	6.7	16.4	20.6 ^c,d^	25.2
Costa Rica	798	68.5	33.6	45.1	64.6 ^e^	85.7	14.7	5.6	10.8	14.3 ^d^	18.2	25.2	8.8	19.5	25.2 ^b,e^	31.3
Ecuador	800	56.2	23.7	39.2	53.0 ^b,d^	68.9	10.3	3.8	7.6	10.0 ^e^	12.2	18.6	6.1	14.4	18.2 ^f^	22.5
Peru	1113	70.4	29.9	49.9	66.5 ^e^	87.6	13.4	4.5	10.3	13.2 ^f^	16.3	20.5	6.7	15.8	20.1 ^d^	24.9
Venezuela	1132	67.0	30.6	44.3	61.7 ^e^	84.9	14.0	5.1	10.5	13.8 ^d,f^	17.2	26.2	8.5	20.2	26.0 ^e^	32.1
**All**	**9218**	**65.5**	**36.5**	**41.1**	**59.4**	**82.5**	**13.2**	**5.8**	**9.2**	**12.6**	**16.5**	**23.9**	**9.7**	**17.1**	**23.1**	**30.0**

g/day: grams per day; %TE: % of the total energy intake; %CHO: % of the total carbohydrates; SD: standard deviation; P25: 25th percentile; P50: 50th percentile (median); P75: 75th percentile. ^1^ Sugar intakes were adjusted by intra-individual variation. ^2^ Within a column, countries followed by different lower-case letters are significantly different according to Kruskal–Wallis test (*α* = 5%).

**Table 3:** Total sugar intakes ^1^ in individuals residing in urban areas of Latin American countries, according to gender; Latin American Health and Nutrition Study (ELANS), 2015.

Country	Gender	*n*	Total Sugar Intake														
			g/Day					%TE					%CHO				
			Mean	SD	P25	P50 ^2^	P75	Mean	SD	P25	P50 ^2^	P75	Mean	SD	P25	P50 ^2^	P75
Argentina	Male	573	126.9	55.2	86.0	123.9 ^a^	166.0	20.5	7.0	15.8	20.6 ^a^	25.1	39.8	11.0	33.3	40.6 ^a^	47.2
	Female	693	105.6	49.4	70.9	97.7 ^b^	129.3	21.6	7.2	16.5	21.1 ^a^	25.7	40.7	10.6	33.4	40.8 ^a^	48.3
Brazil	Male	942	91.2	42.4	61.1	85.4 ^a^	114.3	17.8	6.5	13.3	17.6 ^a^	21.8	35.0	10.6	28.1	35.2 ^a^	42.5
	Female	1058	81.8	35.8	56.3	78.2 ^b^	101.9	20.2	6.4	16.0	20.0 ^b^	24.2	38.8	10.0	32.6	39.1 ^b^	45.8
Chile	Male	425	94.3	35.4	69.3	92.8 ^a^	113.0	19.2	5.1	15.6	19.2 ^a^	22.6	35.5	8.1	30.5	35.7 ^a^	40.9
	Female	454	76.2	29.8	55.5	70.7 ^b^	92.3	20.4	5.9	16.5	20.4 ^b^	23.9	37.1	8.8	31.8	37.2 ^b^	42.4
Colombia	Male	603	114.9	37.6	88.4	110.4 ^a^	135.3	20.1	4.6	17.0	19.9 ^a^	23.0	37.2	7.0	32.5	37.4 ^a^	41.7
	Female	627	104.9	30.3	83.7	101.9 ^b^	124.0	21.8	4.8	18.4	21.4 ^b^	24.9	39.5	7.0	34.3	39.7 ^b^	44.4
Costa Rica	Male	394	104.3	42.1	76.4	100.7 ^a^	124.0	19.5	5.9	15.4	19.3 ^a^	23.2	33.7	9.0	27.6	33.8 ^a^	39.7
	Female	404	87.5	34.0	63.7	81.8 ^b^	105.8	21.8	6.0	17.9	21.8 ^b^	25.0	37.2	8.8	31.9	37.5 ^b^	42.8
Ecuador	Male	397	110.7	35.6	88.2	106.9 ^a^	126.1	18.4	4.6	15.2	18.3 ^a^	20.9	33.6	7.2	29.1	33.5 ^a^	38.4
	Female	403	94.2	32.7	72.6	90.0 ^b^	111.2	19.0	4.6	15.5	18.8 ^a^	22.1	34.1	6.9	29.2	34.5 ^a^	38.5
Peru	Male	523	113.3	37.9	85.9	109.2 ^a^	135.1	19.5	4.8	15.9	19.3 ^a^	22.7	29.6	7.0	24.5	29.5 ^a^	34.9
	Female	590	100.2	30.7	79.6	96.1 ^b^	116.7	21.2	4.7	18.0	21.1 ^b^	24.2	32.5	6.7	27.9	32.3 ^b^	37.2
Venezuela	Male	552	102.9	41.8	72.8	96.4 ^a^	126.9	19.9	5.8	15.8	19.7 ^a^	23.7	37.6	8.8	31.4	38.0 ^a^	43.9
	Female	580	94.9	35.9	68.6	90.9 ^b^	117.2	21.5	5.8	17.6	21.6 ^b^	25.1	39.6	8.8	34.3	39.9 ^b^	46.1
**All**	**Male**	**4409**	**106.4**	**43.5**	**76.0**	**101.7 ^a^**	**129.7**	**19.3**	**5.8**	**15.4**	**19.2 ^a^**	**22.9**	**35.4**	**9.4**	**29.2**	**35.5 ^a^**	**41.5**
	**Female**	**4809**	**93.1**	**37.4**	**67.5**	**89.3 ^b^**	**112.8**	**20.9**	**5.9**	**17.0**	**20.8 ^b^**	**24.5**	**37.8**	**9.2**	**31.7**	**37.8 ^b^**	**43.6**

g/day: grams per day; %TE: % of the total energy intake; %CHO: % of the total carbohydrates; SD: standard deviation; P25: 25th percentile; P50: 50th percentile (median); P75: 75th percentile. ^1^ Sugar intakes were adjusted by intra-individual variation. ^2^ Within a column, in the same country, gender followed by different lower-case letters are significantly different according to Mann-Whitney test (*α* = 5%).

**Table 4:** Added sugar intakes ^1^ in individuals residing in urban areas of Latin American countries, according to gender; Latin American Health and Nutrition Study (ELANS), 2015.

Country	Gender	*n*	Added Sugar Intake (from Total Sugar)														
			g/Day					%TE					%CHO				
			Mean	SD	P25	P50 ^2^	P75	Mean	SD	P25	P50 ^2^	P75	Mean	SD	P25	P50 ^2^	P75
Argentina	Male	573	104.0	58.4	62.1	97.1 ^a^	138.9	16.5	7.3	11.7	16.1 ^a^	21.6	31.9	12.0	23.7	32.3 ^a^	40.6
	Female	693	80.9	50.0	45.4	70.2 ^b^	103.9	16.3	7.7	10.8	15.4 ^a^	20.9	30.4	12.3	21.1	30.2 ^b^	38.5
Brazil	Male	942	61.8	36.5	35.0	56.6 ^a^	82.5	12.0	6.0	7.6	11.4 ^a^	15.6	23.4	10.1	15.9	23.1 ^a^	30.1
	Female	1058	53.9	31.3	29.9	49.8 ^b^	70.5	13.1	6.1	8.8	13.0 ^b^	16.9	25.1	10.1	18.0	25.4 ^b^	32.1
Chile	Male	425	60.2	29.9	38.8	56.0 ^a^	77.7	12.2	4.8	8.8	11.8 ^a^	15.5	22.5	8.3	16.9	22.7 ^a^	28.6
	Female	454	45.0	24.8	27.7	41.5 ^b^	59.3	11.9	5.5	7.8	11.8 ^a^	15.6	21.5	9.1	15.4	21.3 ^a^	28.0
Colombia	Male	603	62.6	26.2	45.9	59.2 ^a^	76.7	11.0	4.0	8.2	10.8 ^a^	13.7	20.4	6.7	15.5	20.2 ^a^	24.9
	Female	627	56.5	21.6	40.8	54.8 ^b^	68.4	11.8	4.0	9.2	11.5 ^b^	14.2	21.3	6.6	17.2	21.0 ^b^	25.7
Costa Rica	Male	394	75.6	36.2	49.7	71.8 ^a^	93.7	14.1	5.6	10.3	14.0 ^a^	17.8	24.4	8.8	18.8	24.5 ^a^	30.4
	Female	404	61.5	29.3	41.3	57.3 ^b^	76.3	15.3	5.6	11.6	14.8 ^b^	18.6	26.0	8.7	20.3	25.8 ^b^	32.0
Ecuador	Male	397	63.3	24.9	45.6	60.4 ^a^	76.7	10.6	3.8	8.0	10.2 ^a^	12.7	19.2	6.2	15.0	18.8 ^a^	23.4
	Female	403	49.3	20.2	35.7	46.6 ^b^	60.3	10.0	3.7	7.5	9.7 ^b^	11.9	17.9	6.0	13.7	17.6 ^b^	21.6
Peru	Male	523	76.9	33.2	53.1	72.6 ^a^	95.3	13.2	4.6	9.9	13.0 ^a^	16.2	20.0	6.9	15.2	19.8 ^a^	24.2
	Female	590	64.7	25.2	47.6	62.3 ^b^	79.4	13.6	4.4	10.6	13.5 ^b^	16.4	20.9	6.6	16.3	20.7 ^b^	25.3
Venezuela	Male	552	69.8	32.3	44.9	66.0 ^a^	89.6	13.5	5.0	10.0	13.3 ^a^	16.7	25.5	8.5	19.5	25.3 ^a^	31.6
	Female	580	64.2	28.6	43.5	59.6 ^b^	80.0	14.5	5.1	11.1	14.4 ^b^	17.6	26.8	8.6	21.2	26.4 ^b^	32.6
**All**	**Male**	**4409**	**71.4**	**39.1**	**45.0**	**65.4 ^a^**	**89.8**	**12.8**	**5.6**	**8.9**	**12.3 ^a^**	**16.1**	**23.6**	**9.6**	**16.8**	**22.8 ^a^**	**29.6**
	**Female**	**4809**	**60.1**	**33.0**	**37.8**	**55.1 ^b^**	**75.7**	**13.4**	**5.8**	**9.4**	**12.8 ^b^**	**16.8**	**24.2**	**9.7**	**17.4**	**23.4 ^b^**	**30.3**

g/day: grams per day; %TE: % of the total energy intake; %CHO: % of the total carbohydrates; SD: standard deviation; P25: 25th percentile; P50: 50th percentile (median); P75: 75th percentile. ^1^ Sugar intakes were adjusted by intra-individual variation. ^2^ Within a column, in the same country, gender followed by different lower-case letters are significantly different according to Mann-Whitney test (*α* = 5%).

**Table 5:** Total sugar intakes ^1^ in individuals residing in urban areas of Latin American countries, according to socioeconomic level; Latin American Health and Nutrition Study (ELANS), 2015.

Country	SEL	*n*	Total Sugar Intake														
			g/Day					%TE					%CHO				
			Mean	SD	P25	P50 ^2^	P75	Mean	SD	P25	P50 ^2^	P75	Mean	SD	P25	P50 ^2^	P75
Argentina	High	65	120.2	52.1	87.5	110.4 ^a^	149.2	21.6	6.9	17.9	20.1 ^a^	25.5	41.2	10.9	33.7	39.5 ^a^	49.6
	Middle	585	114.5	52.3	74.3	109.6 ^a^	145.1	21.1	6.9	16.4	21.1 ^a^	25.2	40.6	10.8	33.5	41.3 ^a^	48.0
	Low	616	115.4	54.3	76.1	105.6 ^a^	145.9	21.1	7.4	16.1	20.7 ^a^	25.8	40.0	10.8	33.0	40.2 ^a^	47.5
Brazil	High	169	100.8	44.7	71.3	94.9 ^a^	119.3	21.2	6.6	16.9	20.2 ^a^	25.1	40.9	9.5	35.6	40.6 ^a^	47.7
	Middle	915	87.6	36.8	61.3	83.6 ^b^	108.6	19.2	6.4	14.7	19.0 ^b^	23.7	37.5	10.3	31.0	37.8 ^b^	44.8
	Low	916	82.2	40.0	54.4	76.0 ^c^	102.8	18.5	6.6	13.9	18.3 ^b^	22.5	35.8	10.6	28.8	36.5 ^c^	42.6
Chile	High	80	78.3	27.5	57.4	78.9 ^a^	96.5	19.4	5.0	15.1	19.7 ^a^	22.7	36.6	8.5	30.8	35.7 ^a,b^	41.3
	Middle	388	83.9	31.0	60.6	80.5 ^a^	102.4	20.1	5.6	16.2	20.0 ^a^	23.4	37.1	8.7	31.7	37.1 ^a^	42.5
	Low	411	87.2	37.3	61.3	79.9 ^a^	107.7	19.7	5.6	16.0	19.4 ^a^	23.4	35.6	8.3	29.9	35.9 ^b^	40.6
Colombia	High	67	119.4	35.3	93.0	119.0 ^a^	140.8	22.0	5.2	18.9	20.5 ^a^	25.4	41.1	8.1	36.5	40.1 ^a^	47.8
	Middle	384	110.2	31.3	87.2	108.5 ^a,b^	129.9	20.6	4.6	17.5	20.6 ^a^	23.0	38.4	6.6	33.8	38.5 ^a,b^	43.1
	Low	779	108.8	35.7	84.8	102.7 ^b^	127.1	21.0	4.8	17.5	20.8 ^a^	24.2	38.1	7.2	33.1	38.3 ^b^	43.0
Costa Rica	High	108	100.2	35.8	75.2	100.1 ^a^	116.8	21.2	5.7	18.0	21.0 ^a,b^	24.2	37.4	8.6	32.4	36.9 ^a^	43.4
	Middle	428	99.2	39.5	72.6	92.3 ^a^	117.3	21.1	5.9	16.9	21.0 ^a^	24.9	36.2	8.8	30.1	36.7 ^a^	42.5
	Low	262	88.6	38.8	60.2	83.8 ^b^	110.7	19.9	6.4	15.8	19.7 ^b^	23.3	33.5	9.4	28.4	33.2 ^b^	39.0
Ecuador	High	104	113.3	41.0	82.7	106.9 ^a^	130.5	20.3	4.6	16.7	20.3 ^a^	22.8	37.4	7.2	32.4	38.1 ^a^	41.8
	Middle	297	104.0	34.1	80.7	98.4 ^a,b^	120.5	18.7	4.4	15.5	18.5 ^b^	21.2	33.9	6.9	29.2	34.0 ^b^	38.4
	Low	399	98.4	33.6	74.7	94.7 ^b^	117.3	18.3	4.7	15.1	18.2 ^b^	21.6	32.8	6.9	28.0	32.9 ^b^	37.5
Peru	High	225	106.2	33.7	84.4	100.8 ^a^	124.6	20.7	4.7	17.8	21.1 ^a^	23.7	33.0	7.3	28.3	33.6 ^a^	37.8
	Middle	355	107.4	33.9	82.8	104.7 ^a^	128.7	20.7	4.6	17.8	20.4 ^a,b^	23.7	31.7	6.5	27.6	31.6 ^a^	36.2
	Low	533	105.7	36.0	80.6	100.6 ^a^	125.1	20.0	5.0	16.3	19.7 ^b^	23.2	30.0	6.9	25.0	29.8 ^b^	34.9
Venezuela	High	62	115.1	37.6	83.5	111.4 ^a^	145.1	22.6	5.1	20.0	22.0 ^a^	25.6	41.5	7.0	37.2	41.6 ^a^	46.1
	Middle	190	102.6	43.4	72.7	99.4 ^b^	122.1	21.0	5.9	17.4	21.0 ^a,b^	24.3	39.1	8.6	33.5	39.0 ^a,b^	46.0
	Low	880	96.9	38.0	69.5	91.4 ^b^	119.8	20.5	5.9	16.6	20.5 ^b^	24.4	38.3	9.0	32.4	38.6 ^b^	44.6
**All**	**High**	**880**	**105.4**	**40.0**	**79.2**	**101.8 ^a^**	**124.5**	**21.0**	**5.5**	**17.4**	**20.6 ^a^**	**24.0**	**37.7**	**8.9**	**32.0**	**37.6 ^a^**	**43.1**
	**Middle**	**3542**	**99.6**	**40.4**	**71.4**	**94.7 ^b^**	**121.6**	**20.2**	**5.9**	**16.3**	**20.1 ^b^**	**23.8**	**37.1**	**9.4**	**31.0**	**37.1 ^a^**	**43.4**
	**Low**	**4796**	**98.2**	**41.5**	**69.8**	**93.1 ^b^**	**119.5**	**19.9**	**6.0**	**15.9**	**19.7 ^b^**	**23.7**	**36.1**	**9.4**	**29.9**	**36.2 ^b^**	**42.2**

SEL: socioeconomic level; g/day: grams per day; %TE: % of the total energy intake; %CHO: % of the total carbohydrates; SD: standard deviation; P25: 25th percentile; P50: 50th percentile (median); P75: 75th percentile. ^1^ Sugar intakes were adjusted by intra-individual variation. ^2^ Within a column, in the same country, socioeconomic level followed by different lower-case letters are significantly different according to Kruskal-Wallis test (*α* = 5%).

**Table 6:** Added sugar intakes ^1^ in individuals residing in urban areas of Latin American countries, according to socioeconomic level; Latin American Health and Nutrition Study (ELANS), 2015.

Country	SEL	*n*	Added Sugar Intake (from Total Sugar)														
			g/Day					%TE					%CHO				
			Mean	SD	P25	P50 ^2^	P75	Mean	SD	P25	P50 ^2^	P75	Mean	SD	P25	P50 ^2^	P75
Argentina	High	65	89.8	52.1	45.4	82.9 ^a^	126.3	15.8	7.2	10.9	15.5 ^a^	19.9	30.1	12.1	21.1	29.4 ^a^	39.6
	Middle	585	88.3	52.6	46.1	81.2 ^a^	121.7	15.9	7.5	10.4	15.5 ^a^	20.7	30.3	12.6	20.8	31.0 ^a^	39.3
	Low	616	94.5	57.7	56.4	82.5 ^a^	124.3	16.9	7.5	11.7	16.2 ^a^	21.6	31.8	11.8	23.6	31.5 ^a^	39.9
Brazil	High	169	66.6	40.7	35.3	62.1 ^a^	85.1	13.6	6.7	8.2	13.7 ^a^	16.9	26.0	10.6	18.4	25.6 ^a^	32.9
	Middle	915	58.4	32.6	34.0	52.9 ^a^	79.1	12.7	6.0	8.0	12.2 ^a,b^	16.6	24.6	10.4	16.9	24.8 ^a,b^	32.0
	Low	916	55.2	33.9	30.1	50.0 ^b^	70.6	12.3	5.9	8.0	11.9 ^b^	15.9	23.6	9.8	16.6	23.5 ^b^	30.0
Chile	High	80	46.4	22.0	31.1	46.2 ^a,b^	60.5	11.4	4.4	8.4	11.0 ^a^	14.6	21.4	7.7	16.9	21.1 ^a^	26.5
	Middle	388	49.8	26.1	30.5	46.5 ^a^	64.6	11.8	5.2	8.2	11.5 ^a^	15.1	21.7	8.7	15.6	21.6 ^a^	28.0
	Low	411	55.9	31.1	32.2	51.6 ^b^	74.7	12.4	5.4	8.4	12.4 ^a^	16.1	22.4	8.8	16.2	22.7 ^a^	28.5
Colombia	High	67	61.9	25.5	45.2	59.6 ^a^	76.9	11.4	4.4	8.7	10.5 ^a^	14.0	21.1	7.1	16.6	20.6 ^a^	25.5
	Middle	384	59.1	22.5	42.0	56.9 ^a^	73.7	11.1	3.7	8.4	11.0 ^a^	13.6	20.6	6.5	16.0	20.5 ^a^	25.0
	Low	779	59.5	24.8	42.8	56.4 ^a^	72.7	11.6	4.1	8.9	11.3 ^a^	13.9	20.9	6.7	16.5	20.7 ^a^	25.2
Costa Rica	High	108	69.9	33.1	48.0	65.4 ^a,b^	87.4	14.6	5.2	11.5	14.0 ^a^	17.7	25.8	8.6	20.7	25.2 ^a^	31.6
	Middle	428	70.6	33.6	46.2	66.0 ^a^	88.7	14.9	5.4	10.9	14.5 ^a^	18.3	25.6	8.5	19.6	25.5 ^a^	31.4
	Low	262	64.5	33.7	40.7	59.4 ^b^	81.4	14.5	6.1	10.6	14.3 ^a^	18.5	24.3	9.3	18.5	24.3 ^a^	30.5
Ecuador	High	104	60.2	29.8	38.9	54.9 ^a^	76.0	10.6	4.1	7.5	10.3 ^a^	12.4	19.6	6.8	14.7	19.5 ^a^	23.2
	Middle	297	57.1	22.1	42.2	53.9 ^a^	69.6	10.2	3.3	7.8	10.2 ^a^	12.2	18.6	5.6	14.7	18.4 ^a^	22.6
	Low	399	54.6	23.1	37.8	51.9 ^a^	67.3	10.2	3.9	7.5	9.7 ^a^	12.2	18.3	6.3	13.9	17.7 ^a^	21.9
Peru	High	225	68.8	30.9	49.8	65.7 ^a^	85.2	13.2	4.6	10.1	13.4 ^a^	16.2	21.0	7.4	16.2	21.2 ^a^	25.9
	Middle	355	72.4	30.5	51.4	69.1 ^a^	88.6	13.8	4.5	10.7	13.5 ^a^	16.7	21.2	6.6	16.4	21.0 ^a^	26.0
	Low	533	69.8	29.0	49.1	66.3 ^a^	88.1	13.2	4.5	10.1	13.0 ^a^	15.9	19.8	6.5	15.5	19.5 ^b^	24.0
Venezuela	High	62	74.9	29.9	51.0	72.1 ^a^	99.2	14.6	4.5	11.3	14.6 ^a^	17.5	26.7	7.0	22.0	27.2 ^a^	30.9
	Middle	190	69.4	32.9	44.3	65.3 ^a,b^	89.0	14.2	5.3	10.8	14.2 ^a^	17.0	26.5	8.6	21.2	25.9 ^a^	32.2
	Low	880	65.9	30.0	43.9	60.3 ^b^	84.2	13.9	5.1	10.4	13.7 ^a^	17.3	26.1	8.6	20.0	25.9 ^a^	32.1
**All**	**High**	**880**	**66.9**	**35.2**	**43.6**	**62.6 ^a^**	**84.4**	**13.1**	**5.5**	**9.4**	**12.9 ^a^**	**16.4**	**23.5**	**9.1**	**17.1**	**22.8 ^a^**	**29.2**
	**Middle**	**3542**	**65.8**	**36.5**	**40.9**	**59.9 ^a^**	**83.9**	**13.2**	**5.9**	**9.0**	**12.6 ^a^**	**16.6**	**24.2**	**9.9**	**17.1**	**23.4 ^a^**	**30.4**
	**Low**	**4796**	**65.0**	**36.7**	**40.9**	**58.8 ^a^**	**81.2**	**13.1**	**5.7**	**9.2**	**12.6 ^a^**	**16.4**	**23.7**	**9.6**	**17.1**	**22.9 ^a^**	**29.7**

SEL: socioeconomic level; g/day: grams per day; %TE: % of the total energy intake; %CHO: % of the total carbohydrates; SD: standard deviation; P25: 25th percentile; P50: 50th percentile (median); P75: 75th percentile. ^1^ Sugar intakes were adjusted by intra-individual variation. ^2^ Within a column, in the same country, socioeconomic level followed by different lower-case letters are significantly different according to Kruskal-Wallis test (*α* = 5%).

**Table 7:** Total sugar intakes ^1^ in individuals residing in urban areas of Latin American countries, according to age groups; Latin American Health and Nutrition Study (ELANS), 2015.

Country	Age Group (Years)	*n*	Total Sugar Intake														
			g/Day					%TE					%CHO				
			Mean	SD	P25	P50 ^2^	P75	Mean	SD	P25	P50 ^2^	P75	Mean	SD	P25	P50 ^2^	P75
Argentina	15–19	152	131.1	52.2	93.1	128.4 ^a^	166.6	21.9	6.3	17.7	21.1 ^a^	26.1	41.8	9.2	34.8	41.5 ^a^	48.1
	20–34	446	120.9	54.0	81.4	110.3 ^a,b^	151.6	21.7	6.9	16.8	21.4 ^a^	25.5	41.3	10.4	35.3	41.6 ^a^	47.8
	35–49	379	112.6	54.6	71.5	106.2 ^b,c^	140.5	20.8	7.4	15.5	20.7 ^a^	25.4	39.8	11.2	32.0	40.1 ^a,b^	48.2
	50–65	289	101.6	46.9	67.2	93.4 ^c^	129.7	20.2	7.4	15.3	20.1 ^a^	24.8	38.6	11.3	30.9	39.4 ^b^	46.9
Brazil	15–19	235	104.1	45.4	72.1	99.5 ^a^	126.9	20.5	5.7	16.7	20.1 ^a^	23.9	39.2	9.2	33.0	39.3 ^a^	44.7
	20–34	745	90.1	40.1	63.3	86.1 ^b^	111.6	19.0	6.5	14.7	18.8 ^b^	23.1	36.9	10.3	30.4	37.3 ^b^	43.9
	35–49	608	83.4	36.9	57.4	78.1 ^c^	104.6	18.9	6.7	14.1	18.8 ^b^	23.4	36.9	10.9	29.3	37.4 ^b^	44.1
	50–65	412	73.2	32.0	50.8	69.6 ^d^	92.3	18.6	6.9	13.6	18.4 ^b^	22.9	36.0	10.6	29.5	36.7 ^b^	43.6
Chile	15–19	118	95.4	31.0	73.2	94.5 ^a^	108.7	21.4	4.5	18.7	21.7 ^a^	24.5	38.6	7.0	33.9	39.3 ^a^	42.6
	20–34	307	90.3	36.2	65.5	86.7 ^a^	109.9	20.0	5.5	16.5	20.0 ^b^	23.5	36.8	8.6	31.8	37.1 ^a,b^	42.3
	35–49	252	81.6	33.4	59.5	75.3 ^b^	98.3	19.2	5.7	15.5	18.9 ^b^	22.3	35.8	8.8	30.1	35.0 ^b^	41.3
	50–65	202	74.8	29.0	52.6	69.0 ^b^	93.2	19.2	5.8	15.0	19.0 ^b^	22.9	35.0	8.7	29.2	35.1 ^b^	40.4
Colombia	15–19	148	118.0	35.2	96.4	114.3 ^a^	136.7	21.0	4.8	17.7	20.7 ^a^	23.9	39.0	6.9	35.1	39.0 ^a^	42.7
	20–34	445	111.1	32.7	87.7	107.0 ^a,b^	132.0	20.7	4.7	17.5	20.3 ^a^	23.5	38.6	6.9	33.7	38.5 ^a^	43.1
	35–49	335	108.7	31.9	85.4	105.1 ^b,c^	126.4	21.1	4.9	17.6	20.8 ^a^	24.2	38.2	7.2	33.1	38.5 ^a^	43.3
	50–65	302	105.0	38.2	78.4	101.3 ^c^	123.7	21.1	4.9	17.9	20.9 ^a^	23.9	37.9	7.4	32.8	37.9 ^a^	42.9
Costa Rica	15–19	121	102.0	35.8	74.4	97.4 ^a,b^	124.6	21.2	5.8	17.1	21.2 ^a^	24.9	36.4	9.3	30.4	36.0 ^a^	43.3
	20–34	301	102.0	40.2	73.1	99.3 ^a^	122.4	20.3	6.1	16.5	20.2 ^a^	23.8	35.8	9.3	30.0	35.6 ^a^	42.2
	35–49	224	94.5	42.4	65.3	87.1 ^b^	112.0	20.8	6.0	16.6	20.6 ^a^	24.6	35.0	8.6	29.0	35.2 ^a^	39.9
	50–65	152	80.5	28.6	60.2	77.4 ^c^	97.8	20.8	6.2	16.9	20.9 ^a^	23.6	34.8	9.3	29.6	34.3 ^a^	40.6
Ecuador	15–19	128	105.7	34.9	80.7	102.8 ^a^	122.2	18.8	4.6	15.4	18.8 ^a^	21.8	33.8	7.0	29.1	34.1 ^a^	38.8
	20–34	316	106.4	35.2	83.2	101.8 ^a^	122.8	18.3	4.4	15.3	18.1 ^a^	21.2	33.5	6.8	29.4	33.9 ^a^	38.0
	35–49	222	100.0	34.0	74.7	96.8 ^a,b^	117.4	18.8	4.8	15.4	18.6 ^a^	22.0	34.0	7.4	28.9	34.0 ^a^	38.9
	50–65	134	93.9	35.5	67.6	91.2 ^b^	109.1	19.2	4.8	15.7	18.8 ^a^	21.9	34.1	7.2	29.0	34.5 ^a^	38.3
Peru	15–19	165	110.4	37.0	84.4	104.3 ^a^	129.8	20.2	4.6	16.3	20.1 ^a^	23.1	30.7	6.7	25.8	30.9 ^a^	35.2
	20–34	460	109.2	33.1	85.7	105.9 ^a^	129.6	20.2	4.7	16.9	20.1 ^a^	23.3	31.2	6.9	26.3	31.0 ^a^	36.0
	35–49	294	104.1	36.3	78.6	100.6 ^a,b^	124.1	20.3	4.9	16.8	20.4 ^a^	23.6	30.9	7.1	26.1	31.1 ^a^	36.3
	50–65	194	99.7	33.7	75.3	92.6 ^b^	117.6	21.0	5.1	17.5	20.7 ^a^	24.5	31.7	7.2	26.5	31.4 ^a^	36.9
Venezuela	15–19	156	104.8	40.3	76.7	97.7 ^a^	131.6	20.3	5.7	16.4	19.9 ^a^	24.3	38.7	8.5	32.6	39.3 ^a^	44.8
	20–34	459	102.9	41.3	71.4	98.0 ^a^	127.3	20.8	5.8	16.8	21.0 ^a^	24.3	39.0	8.8	33.2	39.2 ^a^	45.5
	35–49	313	94.9	36.3	69.4	90.4 ^b^	116.5	20.6	5.8	16.8	20.8 ^a^	24.5	38.2	8.9	33.1	38.7 ^a^	44.5
	50–65	204	91.1	35.6	66.2	85.4 ^b^	112.5	20.9	6.1	17.1	20.7 ^a^	24.8	38.1	9.1	31.7	38.1 ^a^	43.6
**All**	**15–19**	**1223**	**109.2**	**41.4**	**79.9**	**103.4 ^a^**	**131.1**	**20.7**	**5.4**	**16.9**	**20.3 ^a^**	**24.2**	**37.4**	**8.8**	**31.7**	**37.4 ^a^**	**43.2**
	**20–34**	**3479**	**103.5**	**41.2**	**75.8**	**99.0 ^b^**	**125.1**	**20.1**	**5.8**	**16.3**	**20.0 ^b^**	**23.6**	**36.8**	**9.3**	**30.7**	**36.8 ^a,b^**	**42.9**
	**35–49**	**2627**	**96.7**	**40.7**	**68.3**	**91.6 ^c^**	**117.9**	**20.0**	**6.1**	**15.9**	**19.8 ^b^**	**23.8**	**36.5**	**9.6**	**30.0**	**36.4 ^b^**	**42.8**
	**50–65**	**1889**	**89.5**	**38.1**	**62.7**	**84.1 ^d^**	**110.4**	**20.0**	**6.2**	**16.0**	**20.0 ^b^**	**23.8**	**36.1**	**9.5**	**30.0**	**36.3 ^b^**	**42.2**

g/day: grams per day; %TE: % of the total energy intake; %CHO: % of the total carbohydrates; SD: standard deviation; P25: 25th percentile; P50: 50th percentile (median); P75: 75th percentile. ^1^ Sugar intakes were adjusted by intra-individual variation. ^2^ Within a column, in the same country, age group followed by different lower-case letters are significantly different according to Kruskal-Wallis test (*α* = 5%).

**Table 8:** Added sugar intakes ^1^ in individuals residing in urban areas of Latin American countries, according to age groups; Latin American Health and Nutrition Study (ELANS), 2015.

Country	Age Group (Years)	*n*	Added Sugar Intake (from Total Sugar)														
			g/Day					%TE					%CHO				
			Mean	SD	P25	P50 ^2^	P75	Mean	SD	P25	P50 ^2^	P75	Mean	SD	P25	P50 ^2^	P75
Argentina	15–19	152	106.9	48.5	67.0	96.1 ^a^	137.8	17.8	6.2	13.6	17.0 ^a^	21.9	33.8	9.5	27.2	32.3 ^a^	40.2
	20–34	446	98.2	53.7	58.1	89.4 ^a,b^	128.4	17.4	7.5	12.5	16.9 ^a^	21.7	32.9	12.0	25.8	33.0 ^a^	40.8
	35–49	379	90.8	61.8	47.3	81.2 ^b^	117.8	16.3	7.8	10.3	15.4 ^a^	21.4	30.8	12.5	21.3	31.3 ^a^	39.8
	50–65	289	73.5	46.3	37.8	63.7 ^c^	99.5	14.3	7.4	8.5	13.4 ^b^	19.5	27.0	12.3	17.2	25.7 ^b^	36.3
Brazil	15–19	235	74.2	39.2	47.2	66.2 ^a^	96.6	14.5	5.7	10.5	14.4 ^a^	18.0	27.6	9.5	21.3	27.5 ^a^	33.8
	20–34	745	62.3	34.3	37.3	58.2 ^b^	82.2	13.1	5.9	8.9	12.8 ^b^	16.7	25.3	9.9	18.4	25.1 ^b^	31.7
	35–49	608	55.5	31.9	31.3	50.2 ^c^	73.5	12.5	6.1	7.8	12.2 ^b^	16.4	24.2	10.2	16.1	24.3 ^b^	31.6
	50–65	412	42.7	26.7	22.7	36.8 ^d^	58.1	10.7	5.9	6.4	10.0 ^c^	14.5	20.6	10.0	13.2	20.3 ^c^	27.6
Chile	15–19	118	58.2	25.6	41.1	55.3 ^a^	74.7	12.9	4.4	10.1	12.6 ^a^	15.9	23.2	7.3	18.8	23.5 ^a^	28.5
	20–34	307	57.3	31.1	34.1	51.1 ^a,b^	77.7	12.5	5.3	8.4	12.1 ^a^	16.2	22.9	8.9	16.1	23.0 ^a^	29.5
	35–49	252	50.9	27.9	31.0	46.9 ^b^	63.9	11.9	5.3	8.4	11.4 ^a,b^	14.8	22.1	8.8	16.2	21.4 ^a,b^	27.4
	50–65	202	43.2	23.6	24.8	40.6 ^c^	56.9	11.0	5.2	6.9	11.0 ^b^	14.7	19.9	8.7	13.3	20.1 ^b^	26.7
Colombia	15–19	148	65.0	26.3	48.3	62.4 ^a^	79.7	11.6	4.2	9.0	11.4 ^a^	13.9	21.5	6.8	17.0	22.2 ^a^	26.0
	20–34	445	60.2	22.2	44.0	58.6 ^a,b^	73.9	11.3	3.8	8.8	11.1 ^a^	13.6	21.1	6.4	17.1	20.7 ^a^	25.2
	35–49	335	58.9	23.8	43.4	54.6 ^b,c^	73.9	11.4	4.1	8.5	11.2 ^a^	13.9	20.7	6.8	15.8	20.3 ^a^	25.1
	50–65	302	56.4	25.7	39.3	54.8 ^c^	69.8	11.4	4.2	8.6	11.2 ^a^	13.8	20.4	6.8	16.2	20.2 ^a^	24.7
Costa Rica	15–19	121	75.0	31.3	51.9	72.1 ^a^	91.8	15.6	5.4	11.3	15.1 ^a^	19.2	26.6	8.4	20.0	26.6 ^a^	32.8
	20–34	301	73.4	33.4	49.3	69.3 ^a^	92.7	14.6	5.5	10.6	14.4 ^a,b^	18.1	25.7	8.8	20.3	25.9 ^a^	31.6
	35–49	224	68.8	36.9	44.2	64.7 ^a^	82.2	15.1	5.7	11.2	14.6 ^a,b^	18.4	25.3	8.5	19.1	25.2 ^a,b^	30.8
	50–65	152	53.2	25.5	35.6	50.5 ^b^	69.4	13.7	5.8	9.7	13.2 ^b^	17.1	22.9	9.0	17.5	22.7 ^b^	28.6
Ecuador	15–19	128	59.6	21.4	45.3	55.9 ^a^	68.9	10.8	3.8	8.5	10.2 ^a^	12.3	19.3	6.0	15.1	18.5 ^a,b^	22.7
	20–34	316	60.7	24.3	43.7	57.1 ^a^	73.1	10.5	3.5	7.9	10.3 ^a^	12.2	19.1	5.8	15.0	19.1 ^a^	22.6
	35–49	222	52.9	22.3	36.5	51.0 ^b^	63.7	10.0	3.7	7.4	9.7 ^a^	12.1	18.0	6.2	13.7	17.3 ^a,b^	21.6
	50–65	134	48.1	24.1	31.9	43.1 ^b^	60.6	9.8	4.2	6.6	9.3 ^a^	12.2	17.4	6.8	12.0	16.9 ^b^	22.5
Peru	15–19	165	74.1	28.3	53.8	67.5 ^a^	89.4	13.6	4.2	10.7	13.1 ^a^	16.0	20.7	6.4	16.2	20.3 ^a^	25.2
	20–34	460	72.9	28.5	52.8	70.9 ^a^	89.2	13.5	4.3	10.4	13.3 ^a^	16.2	20.8	6.6	16.3	20.3 ^a^	25.0
	35–49	294	68.8	32.6	47.4	64.9 ^a,b^	85.8	13.2	4.8	9.9	13.1 ^a^	16.4	20.1	6.9	15.5	20.1 ^a^	24.6
	50–65	194	63.8	29.2	44.9	60.2 ^b^	80.1	13.3	4.8	10.0	13.4 ^a^	16.4	20.0	7.0	15.2	19.8 ^a^	25.3
Venezuela	15–19	156	72.2	30.0	51.3	66.2 ^a^	91.3	14.1	4.5	10.9	13.8 ^a^	17.2	26.8	7.5	21.4	26.6 ^a^	32.6
	20–34	459	71.3	32.8	45.3	67.5 ^a^	92.9	14.4	5.3	10.9	14.3 ^a^	17.8	27.0	8.7	21.0	27.1 ^a^	32.9
	35–49	313	63.3	28.4	43.3	58.8 ^b^	78.0	13.8	5.1	10.3	13.5 ^a^	17.1	25.6	8.8	19.3	25.1 ^a,b^	31.7
	50–65	204	58.8	26.5	41.4	54.2 ^b^	73.2	13.6	5.1	10.1	13.1 ^a^	16.7	24.6	8.3	19.0	23.8 ^b^	29.5
**All**	**15–19**	**1223**	**73.9**	**35.9**	**49.9**	**66.4 ^a^**	**91.2**	**13.9**	**5.3**	**10.2**	**13.3 ^a^**	**17.1**	**25.2**	**9.0**	**18.8**	**24.4 ^a^**	**31.2**
	**20–34**	**3479**	**69.6**	**36.3**	**44.7**	**64.2 ^b^**	**87.7**	**13.5**	**5.7**	**9.5**	**13.0 ^b^**	**16.8**	**24.6**	**9.6**	**17.9**	**23.8 ^a^**	**30.7**
	**35–49**	**2627**	**63.9**	**38.2**	**39.6**	**57.0 ^c^**	**79.5**	**13.1**	**5.9**	**8.9**	**12.5 ^c^**	**16.5**	**23.8**	**9.9**	**16.8**	**22.8 ^b^**	**29.9**
	**50–65**	**1889**	**54.8**	**31.9**	**32.5**	**50.1 ^d^**	**69.3**	**12.1**	**5.7**	**8.0**	**11.7 ^d^**	**15.5**	**21.8**	**9.5**	**15.2**	**21.3 ^c^**	**27.6**

g/day: grams per day; %TE: % of the total energy intake; %CHO: % of the total carbohydrates; SD: standard deviation; P25: 25th percentile; P50: 50th percentile (median); P75: 75th percentile. ^1^ Sugar intakes were adjusted by intra-individual variation. ^2^ Within a column, in the same country, age group followed by different lower-case letters are significantly different according to Kruskal-Wallis test (*α* = 5%).
